# Combined Effects of *BEIIb* and *SSIIa* Alleles on Amylose Contents, Starch Fine Structures and Physicochemical Properties of Indica Rice

**DOI:** 10.3390/foods12010119

**Published:** 2022-12-26

**Authors:** Yaqi Hu, Yanni Zhang, Shouwu Yu, Guofu Deng, Gaoxing Dai, Jinsong Bao

**Affiliations:** 1 Institute of Nuclear Agricultural Sciences, College of Agriculture and Biotechnology, Zhejiang University, Zijingang Campus, Hangzhou 310058, China; 2 Hainan Institute of Zhejiang University, Yazhou Bay Science and Technology City, Yazhou District, Sanya 572025, China; 3 Institute of Crops and Nuclear Technology Utilization, Zhejiang Academy of Agricultural Sciences, Hangzhou 310021, China; 4 Rice Research Institute, Guangxi Academy of Agricultural Sciences, Nanning 530007, China

**Keywords:** rice, starch synthase, branching enzyme, starch structure, functional property

## Abstract

Starch branching enzyme IIb (BEIIb) and soluble starch synthase IIa (SSIIa) play important roles in starch biosynthesis in cereals. Deficiency in the BEIIb gene produces the *amylose extender* (*ae*) mutant rice strain with increased amylose content (AC) and changes in the amylopectin structure. The SSIIa gene is responsible for the genetic control of gelatinization temperature (GT). The combined effects of *BEIIb* and *SSIIa* alleles on the AC, fine structures, and physicochemical properties of starches from 12 rice accessions including 10 recombinant inbred lines (RIL) and their two parents were examined in this study. Under the active *BEIIb* background, starches with the *SSIIa*-GC allele showed a higher GT than those with the *SSIIa*-TT allele, resulting from a lower proportion of A chain and a larger proportion of B1 chains in the amylopectin of *SSIIa*-GC. However, starch with the *BEIIb* mutant allele (*be2b*) in combination with any *SSIIa* genotype displayed more amylose long chains, higher amylose content, B2 and B3 chains, and molecular order, but smaller relative crystallinity and proportion of amylopectin A and B1 chains than those with *BEIIb*, leading to a higher GT and lower paste viscosities. These results suggest that *BEIIb* is more important in determining the structural and physicochemical properties than *SSIIa*. These results provide additional insights into the structure-function relationship in indica rice rather than that in japonica rice and are useful for breeding rice with high amylose content and high resistant starch.

## 1. Introduction

The emergence of hybrid rice technology has greatly increased the yield potential of rice and solved the problem of food shortages. However, people’s demand for a better life and tastier rice is increasing with the gradual improvement of people’s living standards. Starch is the most important element of rice, accounting for about 90% of the milled rice on a dry weight basis, and its physicochemical properties mainly determine rice cooking and eating quality (CEQ) [1,2]. Starch is composed of amylose and amylopectin molecules. Amylose is a relatively long linear α-glucan linked by α-(1→4) glycosidic bonds [3]. Amylopectin is a highly branched polymer with linear chains linked with α-(1→4) glycosidic bonds and branch points linked by α-(1→6) glycosidic bonds [3,4]. Starch biosynthesis is a complicated process. Amylose is synthesized under the sole action of the granule-bound starch synthase (GBSS) encoded by the *Waxy* (*Wx*) locus [5]. The synthesis of amylopectin requires a combination of multiple enzymes, including four soluble starch synthase (SS) isoforms (SSI, SSII, SSIII and SSIV), two starch branching enzyme (BE) isoforms (BEI and BEII), and starch debranching enzymes [5].

Natural variations in starch biosynthesizing genes are responsible for the different starch physicochemical properties and CEQ. Although many genes involved in starch biosynthesis have natural variations, the variation in *Wx* and *SSIIa* is well known and has great effects on the improvement in grain quality since they are responsible for the genetic basis of amylose content (AC) and gelatinization temperature (GT), respectively [6]. A G/T single nucleotide polymorphism (SNP) (AGGTAT/ AGTTAT) at 5′ splice site of the first intron of *Wx* pre-mRNA can differentiate indica type *Wx^a^* (G SNP) and japonica type *Wx^b^* (T SNP) alleles [7]. A simple sequence repeat (SSR) or microsatellite, cytosine-thymine (CT)n, in the *Wx* gene can be used to classify rice with different AC classes, which can explain >75% of the variation in AC among various rice accessions [8,9,10]. Two nonsynonymous SNPs, i.e., G/A at the 4198 bp, and GC/TT at the 4329/4330 bp of *SSIIa* have been discovered to affect the GT [11,12,13]. The G/GC combination (or haplotype) rice has an intermediate or high GT, whereas rice with the A/GC or G/TT combination has a low GT, but the A/TT haplotype does not exist in nature [12,13,14,15,16].

Mutations in the starch biosynthesizing genes may lead to enzyme deficiency and starches with altered structure and functionality. The *waxy* or glutinous rice with a characteristic of <2% AC is derived from a common mutation of the *Wx* gene. The knocking out of the *Wx* gene with physical and chemical mutagenesis, or a new tool such as the clustered regularly interspaced short palindromic repeats (CRISPR)/associated protein-9 (Cas9) can easily produce the *waxy* mutant [17]. The *amylose extender* (*ae*) mutant is derived from a defect in the *BEIIb* gene [18,19] that specifically changes the amylopectin structure by decreasing the number of short chains with a degree of polymerization (DP) of 17 or less, with the greatest decrease in the DP8-12 chains [18]. In addition, AC is significantly positively correlated with resistant starch content, which is defined as the sum of starch and products of starch degradation not absorbed in the small intestine of healthy individuals [20]. Therefore, *ae* starch is a kind of resistant-starch resource, which can offer a wide array of health benefits to humans [21].

The combination of alleles of different starch biosynthesizing genes has been reported to change the physicochemical properties and starch fine structures. For example, Kubo et al. [22] indicated that *ae* and *wx/ae* double mutant starches display no difference in the chain-length distribution (CLD) of amylopectin and morphology of the starch granule, but *wx/ae* starch showed a higher pasting temperature and higher peak viscosity. The role of the *Wx* and *SSIIa* combination on the amylose fine structure was reported by Wang et al. [23], who found that *Wx* SNPs can affect AC but they are unable to alter the CLD of both amylopectin and amylose. Itoh, et al. [21] introduced the *SSIIa* from an indica rice cultivar to an *ae* mutant, which led to a higher proportion of amylopectin chains with DP11–18 than those in *be2b,* and the introduction of *Wx* from an indica rice cultivar significantly increased AC in the endosperm starch. Ida et al. [24] found that the AC and starch crystallinity in the japonica *ss2a/be2b* mutant were significantly higher than those in the *be2b* single mutant. Although previous studies have reported the effects of allele combinations of different genes on physical and chemical properties and amylopectin chain length distribution (CLD) [21,22,24], most of these studies were focused on japonica rice. However, there is limited information about the effects of *SSIIa* and *BEIIb* on the AC, starch fine structure, crystal structure, and functional characteristics of indica rice starch.

In this study, we hypothesize that the starch structure-function relationship would be altered in different combinations of *BEIIb* and *SSIIa* genotypes. Indica rice recombinant inbred lines (RIL) with different *BEIIb* and *SSIIa* genotypes were produced by cross-breeding assisted with a selection of molecular markers. The objective of this study is to characterize the structural and functional properties of the RILs and elucidate the effects of *BEIIb* and *SSIIa* genotype combinations on the structure-function relations of starch.

## 2. Materials and Methods

### 2.1. Materials

An *indica* rice cultivar Longtefu B (LTFB) with *SSIIa* allele-TT/*BEIIb* genotype and an *amylose extender (ae)* mutant BP577 with *SSIIa* allele-GC/*be2b* genotype was crossed to obtain F_1_. Each F_2_ breeding line was advanced by the single seed descent method to generate a recombinant inbred line (RIL) population. Molecular markers were applied in the F_4_ generation to select the recombination of *SSIIa* and *BEIIb* alleles. Ten breeding lines (BL01~BL10) were selected and advanced to F_7_. In this study, a completely randomized design with two replications was conducted at the Zhejiang University farm, Hangzhou, China. Mature seeds were harvested in late September.

### 2.2. Genotyping of Breeding Lines

The leaves harvested from seedlings of each RIL were used for genomic DNA isolation with the CTAB method of Doyle [25]. The genotypes of each RIL were analyzed by molecular marker [26,27]. The sequences of primers used in this study are listed in Table S1. The PCR products were separated by running agarose or polyacrylamide gel electrophoresis (PAGE) gels [26,27]. To confirm whether all the rice lines carried the same *Wx* allele, the microsatellites (CT)n were amplified with a primer, as described in Bao et al. [9].

### 2.3. Starch Isolation and Debranching

Starch was extracted from rice flour following the methods of Syahariza, Li, and Hasjim [28]. The purified starch was debranched by the addition of 2.5 µL isoamylase (1000 U/mL) from Pseudomonas sp. (Megazyme International Ltd., Bray, Co. Wicklow, Ireland), and mixed with 100 µL acetate buffer solution (0.1 M, pH 3.5) and 5 µL sodium azide solution (0.04 g/mL), and then incubated at 37 °C for 3 h. The starch solution was neutralized with 100 µL NaOH (0.1 M) and heated at 80 °C for 1 h, then freeze-dried overnight. The debranched starch fraction was dissolved in DMSO/LiBr (5%) for further size-exclusive chromatography (SEC) analysis.

### 2.4. Size-Exclusion Chromatography (SEC)

The chain-length distributions (CLDs) of the debranched starch were separated with the SEC using an LC20AD system (Shimadzu Corporation, Kyoto, Japan) equipped with three columns (pre-column, Gram 100, and Gram 1000) (PSS, Mainz, Germany) in sequence according to the method described in Zhang et al. [26]. The weight CLD of the debranched chains with the degree of polymerization (DP) or *X*, denoted as *w*_de_(log*X*), obtained from the DRI signal, was converted to the corresponding number distribution *N*_de_(*X*) by *w*_de_(log*X*) = *X*^2^ *N*_de_(*X*) (a relation that holds only for linear polymers) [29]. *X_AP1_* and *X_AP2_* are the DP value at the peak 1 & 2 of amylopectin, and *X_AM_* represents the DP value at the amylose peak, *h_AP2/_h_AP1_* is the ratio of the peak heights of amylopectin, and *h_AM_* is the peak height of amylose.

### 2.5. Amylose Content Measurement

The AC was calculated from the SEC weight distributions of debranched starch by dividing the area under the curve (AUC) for 100 < *X* < 10,000 by the whole area, i.e., the whole weight distribution of the debranched starch molecules.

### 2.6. FACE Analysis

The debranched starches were labeled with the fluorescence 8-amino-1,3,6-pyrenetrisulfonic acid according to the method of Wu et al. [30]. The amylopectin chain-length distribution was separated in an MDQ Plus Fluorophore-assisted carbohydrate electrophoresis (FACE) System, coupled with an argon-ion laser as the excitation source and a solid-state laser-induced fluorescence detector. The side chains of amylopectin can be divided into four groups according to their DP: DP ≤ 12 (A chain), 13 ≤ DP ≤ 24 (B1 chain), 25 ≤ DP ≤ 36 (B2 chain), and DP ≥ 37 (B3 and ultra-long chain) [31]. The proportions of the different groups were calculated from the amylopectin CLDs denoted as fa, fb1, fb2, and fb3, respectively.

### 2.7. X-ray Diffraction

X-ray diffraction analysis of the starch granules was conducted on an X-ray powder diffractometer (D8, Bruker, Karlsruhe, Germany), at a voltage of 40 kV and a current of 40 mA. The starches were tightly packed into the glass sample holder, and data were collected over an angular range of 2*θ* from 3° to 40° with a step of 0.05°. The relative crystallinity (RC, %) was calculated using Origin software (OriginLab Co., Northampton, MA, USA) following the method of Zhang, et al. [26].

### 2.8. Attenuated Total Reflectance-Fourier Transform Infrared Spectroscopy

A Varian 7000 Fourier transform infrared spectrometer equipped with a DTGS detector and an ATR single-reflectance cell containing a germanium crystal (45° incidence angle) (PIKE Technologies, Madison, WI, USA) was used to measure the short-range ordered structure of the starch granules [32]. The sample was scanned 64 times from 4000 to 800 cm^−1^ with a resolution of 4 cm^−1^. The relative absorbances at 1045, 1022, and 995 cm^−1^, which represent the ordered regions, amorphous regions, and the bonding in the hydrated carbohydrate helices in starch, respectively [33], were extracted from the deconvoluted spectra and measured from the baseline to the peak height. The 1045/1022 cm^−1^ ratio represents the degree of order in the starch external region, while the 1022/995 cm^−1^ ratio represents an index for the ratio of amorphous to carbohydrate structure starch.

### 2.9. RVA Pasting Viscosity

Three grams of rice flour was weighed in an aluminum can and then mixed with deionized water (25 g). The pasting properties were analyzed using a rapid visco-analyzer (RVA) (Model 4500, Perten Instrument, Hägersten, Sweden) according to the method of Bao et al. [1]. The idle temperature was set to 50 °C, held for 1.0 min, and then linearly ramped up to 95 °C until 4.8 min had elapsed, held at 95 °C until 7.5 min had elapsed, before being linearly ramped down to 50 °C after 11 min had elapsed, and held at this temperature until 12.5 min had elapsed. The RVA trace was analyzed by TCW software to obtain the peak (PV), host paste (HPV), and cold paste (CPV) viscosities. The breakdown, (BD = PV − HPV), consistency (CS = CPV − HPV), and setback (SB = CPV − PV) were derived from the PV, HPV, and CPV. The unit of all viscosity parameters is Rapid Visco Units (RVU).

### 2.10. DSC Thermal (Gelatinization) Properties

The thermal (gelatinization) characteristics were measured by using a DSC 2920 thermal analyzer (TA Instruments, Newcastle, DE, USA) equipped with DSC standard and dual sample cells, according to the method of Bao et al. [34]. Rice flour (2.0 mg, d.b.) was weighed into an aluminum pan to which 6 μL of distilled water was added. The pan was hermetically sealed, equilibrated at room temperature for 1 h, and then heated at a rate of 10 °C/min from 30 °C to 110 °C. A sealed empty pan was used as a reference. Onset (T_o_), peak (T_p_), conclusion (T_c_) temperature, and enthalpy (ΔH) of gelatinization were calculated automatically using the Universal Analysis 2000 program (Version 4.4A) software (TA Instruments, Newcastle, DE, USA).

### 2.11. Statistical Analysis

All analyses were carried out in duplicate and reported as mean ± SD. Analysis of variance (ANOVA) was conducted in SAS 9.0 (SAS Institute Inc., Cary, NC, USA) with Tukey’s pairwise comparisons (*p* < 0.05). Clustering analysis of the genotypes based on the structural and physicochemical properties was conducted using IBM SPSS Statistics 25.

## 3. Results and Discussion

### 3.1. Genotyping of The Breeding Lines

**Figure 1:**
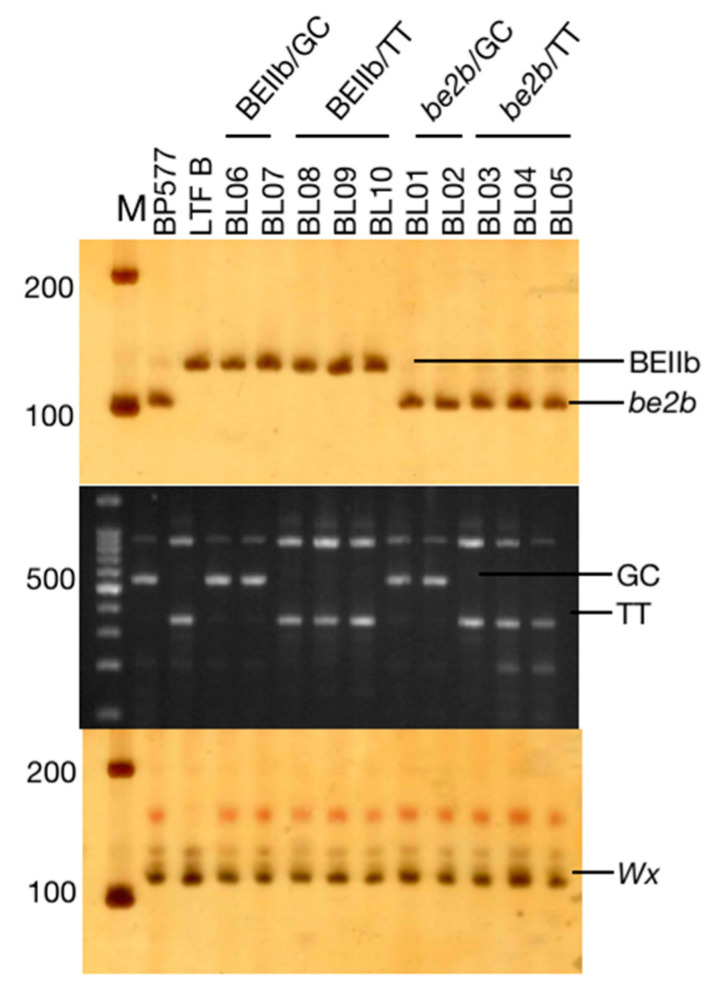
Genotyping of *Wx*, *BEIIb,* and *SSIIa* alleles in RILs.

The use of PAGE gel can easily identify the two alleles of *BEIIb*. The PCR products of *BEIIb* or *be2b* were digested with *MboI* restriction endonuclease (Figure 1), which can separate the *BEIIb* or *be2b* (mutant) allele. Two pairs of primers facing each other were used in a PCR reaction to amplify *SSIIa* GC/TT alleles (Figure 1), which are denoted as *SSIIa*-GC (GC) and *SSIIa*-TT (TT) alleles. To confirm whether all rice carried the same *Wx* allele, the (CT)n microsatellites were amplified, displaying the same allele (Figure 1). The parent BP577 is an *amylose extender (ae)* mutant, so it has the *be2b* allele and also harbors the *SSIIa*–GC allele (Figure 1; Table 1). The parent LTFB has the *BEIIb* and *SSIIa*-TT alleles. Theoretically, there are four combinations of *SSIIa* and *BEIIb* alleles. As a result, four combinations were found in the RILs, including *SSIIa*-GC/*BEIIb*, *SSIIa*-TT/*BEIIb*, *SSIIa*-GC/*be2b*, and *SSIIa*-TT/ *be2b* (Table 1).

### 3.2. SEC Chain-Length Distributions of the Debranched Starch

**Figure 2:**
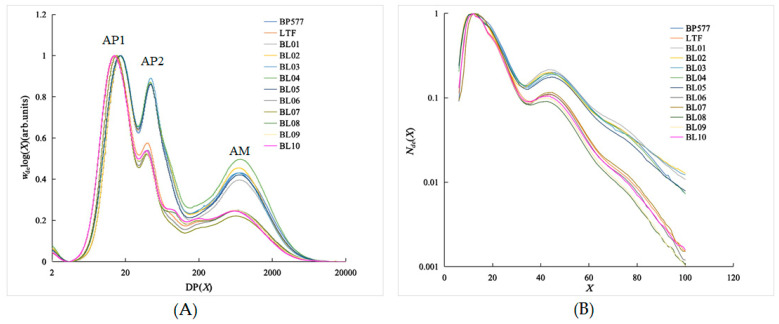
The chain length distribution (CLD) of debranched starch. (**A**): SEC weight CLDs of the whole range of debranched starch; (**B**): FACE number CLDs of debranched amylopectin branches. All distributions are normalized to the global maximum peak.

The SEC weight distribution of debranched starch from the GC/*BEIIb*, TT/*BEIIb*, GC/*be2b*, and TT/*be2b* series is shown in Figure 2A. The maximum SEC weight distribution of each sample is normalized to an arbitrary value of 1. Three peaks exist in the SEC-weight CLDs (Figure 2A). Debranched amylopectin has two peaks: the first peak at DP from 14 to 17, or *X_AP1_*, indicates short amylopectin chains, while the second peak at DP from 38 to 44, or *X_AP2_*, indicates a long amylopectin chain (Table 1; Figure 2A). Debranched amylose has only one peak at DP 580 (LTFB) to 729 (BP577). Two parents showed distinctive CLD parameters, and most of the parameters of their offspring were between two parents with some transgressive segregations that had larger or smaller values than the parents (Table 1). The parent BP577 and the breeding lines with the *be2b* allele mostly had larger *X_AP1_, X_AP2_,* and *X_AM_*, indicating that their amylopectin and amylose had larger molecular sizes than those carrying the *BEIIb* allele. The lines with *SSIIa*-GC/*BEIIb* had slightly larger *X_AP1_* and *X_AP2_* than those with *SSIIa*-TT/*BEIIb*. Rice with the *be2b* allele had much higher *h_AP2_/h_AP1_* than those with the *BEIIb* allele, which is in agreement with the result of Tappiban, et al. [19], who showed that the mutant deficiency in *BEIIb* had the *h_AP2_/h_AP1_* of 0.947. The value of *h_AP2_/h_AP1_* of the *be2b* genotypes was as high as that of potato starches [35], and a little larger than that of cassava starches and common rice starches [23,36].

Both parents carry the same *Wx^a^* allele with high AC, so all the breeding lines had AC larger than 27%. The AC of the rice with the *be2b* allele (32.27%~34.84%) was significantly higher than those with the *BEIIb* allele (26.82%~28.26%). However, there is no significant difference between the *SSIIa* GC and TT alleles (Table 1). In previous reports, the *be2b* allele represents the lack of BEIIb and can increase the AC of rice starches [19,37,38,39] and corn starches [40]. Since GBSS is responsible for amylose synthesis, these results may suggest that the GBSS might be inhibited by an active BEIIb.

The CLD number of the debranched starch from FACE is shown in Figure 2B, and the maximum value is normalized to 1. The maximum peak is at DP ~ 12, and a small bump is found at DP30~40. The amylopectin chains can be divided into four groups according to DP: DP ≤ 12 (A chain), 13 ≤ DP ≤ 24 (B1 chain), 25 ≤ DP ≤ 36 (B2 chain), and DP ≥ 37 (B3 and ultra-long chain) [31]. The proportion of amylopectin CLD showed a significant difference between starches from different *BEIIb* and *SSIIa* genotypes (Table 2), which was in agreement with the results from the SEC weight distribution (Table 1 and Figure 2A). The proportion of A chain and B1 chain of starches with the *be2b* genotype ranged from 20.29% to 21.73% and 41.74% to 44.06%, which was much lower than those with the *BEIIb* genotype. Similarly, the proportions of the B2 and B3 chains of *be2b* were much higher than those of the *BEIIb* allele, leading to the average length of the *be2b* starch amylopectin being significantly higher than that of its *BEIIb* counterpart. The structural changes derived from BEIIb deficiency were in agreement with previous reports [18,21,22,26]. These results confirmed that the *ae* mutant not only prolongs the branch chain length of amylopectin but also significantly increases amylose content in starch [37]. Under the same *BEIIb* allele background, the A chain content of the SSIIa-GC starch was significantly lower, while the B1 chain content was significantly higher than that of SSIIa-TT (Table 2). In common rice accessions, it was proven some time ago that rice with the GC allele has lower fa chain content and fa/fb1 ratio than that with the TT allele [14]. However, under the *be2b* allele background, rice materials with SSIIa-GC starch had a little higher A chain content than those with the SSIIa-TT allele, while the reverse was found for the content of the B1 chain (Table 2).

### 3.3. Crystalline Structure

**Figure 3:**
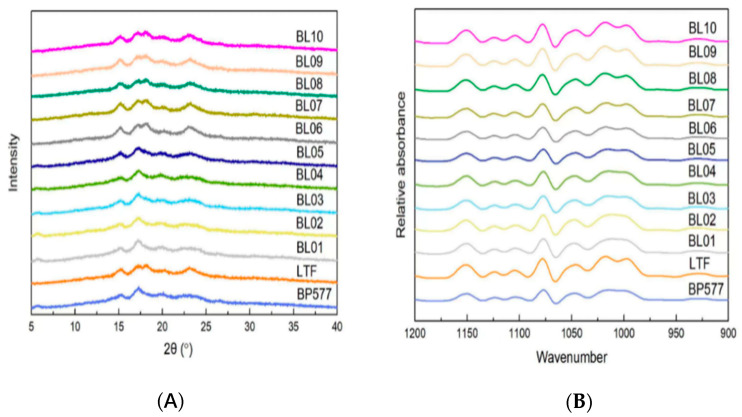
XRD spectra (**A**) and deconvoluted ATR-FTIR spectra (**B**) of rice starches.

The XRD patterns of the two parents and 10 breeding lines are shown in Figure 3A. The A-type crystallinity starch has the characteristic of containing more short branch chains of amylopectin, while the B-type crystallinity has the characteristic of containing more long branch chains, and the C-type starch is a mixture of A and B -type crystallinity. Starches with the *BEIIb* allele had a typical A-type crystalline with four peaks at diffraction angles (2*θ*) of 15°, 17°, 18°, and 23° in common with those of common rice and other cereal starches [19,37]. The BEIIb deficiency mutant starch generally has B-type crystallinity [19,22,24,37,40]. In this study, starches with the *be2b* allele had a C-type pattern with a strong peak at 17° and weak peaks at 5°, 15°, and 23°, which may be derived from incomplete suppression of the expression of the BEIIb [37]. The relative crystallinity (RC) showed significant differences between genotypes with different allele combinations (Table 3). Among starches with A-type crystalline, values of RC with the GC allele (BL06 and BL07) were a little higher than those of the TT allele (BL08-10). Starch with high RC was in alignment with higher B1 chain proportion, so in the common starches, those with the GC allele had higher fb1 content (Table 2), resulting in high RC. However, among the starches with C-type crystallinity, values of RC with the GC allele (BL01-BL02) were a little lower than those of the TT allele (BL03-05) (Table 3). It is possible that the short chains generated in the *BEIIb* mutant were inhibited from elongation due to the action of the SSIIa isoform. It is also suggested that SSIIa forms a heteromeric protein complex with SSI and BEIIb in cereal endosperm to synthesize amylopectin [6,41,42]. Therefore, the lack of BEIIb isoform may decrease the amount of functional protein complex, which results in less short-chain elongation, even though the SSIIa is abundant.

The 900–1200 cm^−1^ region of the FTIR spectra of the two parents and their breeding lines is shown in Figure 3B. The 1045/1022 cm^−1^ ratio displayed significant differences between the rice materials, ranging from 0.634 (BL10) to 0.812 (BL01) (Table 3). The higher ratio of 1045/1022 cm^−1^ may indicate that the starch has a higher degree of ordered structure. In either *BEIIb* or *SSIIa* allele backgrounds, it is clearly shown that starches with the *be2b* or GC allele had a higher degree of ordered structure than their counterparts with the *BEIIb* or TT allele. The ratio 1022/995 cm^−1^ indicates the proportion of amorphous structure. The starches with the *BEIIb* allele had a higher ratio of 1022/995 cm^−1^ than those with the *be2b* allele, which is not in agreement with the relative crystallinity data (Table 3).

### 3.4. Thermal Properties

The onset temperature (T_o_), peak temperature (T_p_), conclusion temperature (T_c_), and the enthalpy of gelatinization (ΔH) of all the rice starches are presented in Table 4. For common starch with the *BEIIb* allele, it is well known that rice accessions with the GC allele had intermediate or high GT, while those with the TT SNP had a low GT [13,14,16]. BL06 and BL07 had a Tp of around 78 °C, and those of BL08-10 had a T_p_ of around 65 °C (Table 4). The difference in gelatinization temperature can be easily explained by the difference in amylopectin CLDs since the fa chain content and fa/fb1 ratio are negatively correlated with GT, whereas fb1 chain content is positively correlated with GT [14]. The ΔH of BL06 and BL07 was the highest among all samples (Table 4), partially because both these samples also had the highest RC (Table 3). It is plausible that the higher fb1 chain content in the BL06 and BL07 starches resulted in their highest ΔH and RC (Table 2). However, among the rice samples with the *be2b* allele, none of the thermal properties displayed a significant difference between the GC and TT alleles (Table 4). However, in japonica rice, Ida et al. [24] indicated that the GT of the *ss2a/be2b* double mutants was higher than that of the WT mutant line but lower than that of the *be2b* mutant lines. The *be2b* allele or the *ae* mutation caused the rice to synthesize a much longer amylopectin chain, leading to a higher gelatinization temperature [18,37]. Furthermore, *BEIIb* may suppress the expression of *SSIIa* to a certain extent, or else, SSIIa function is not so important when BEIIb is defect. Thus, no significant difference between the GC and TT alleles in the *be2b* background was found in the *be2b* background (Table 4).

### 3.5. Pasting Viscosities

**Figure 4:**
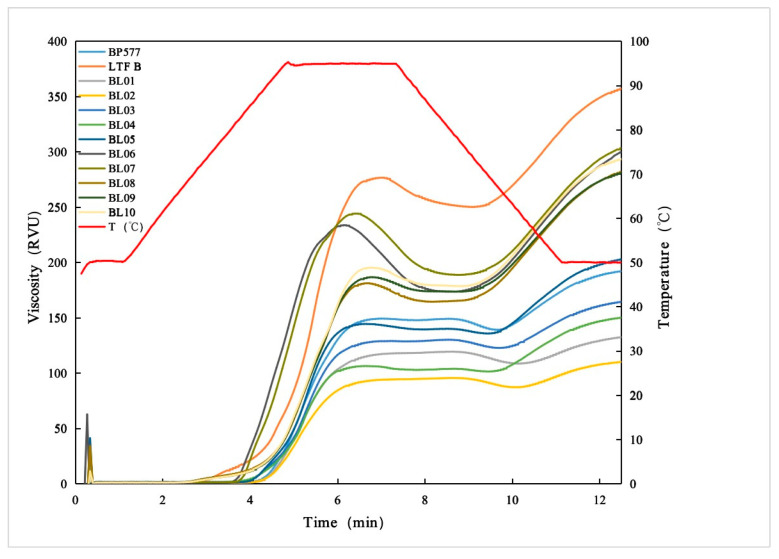
Rapid viscosity profiles of rice starches.

The RVA pasting traces of the rice materials are displayed in Figure 4 and Table 5. For the common rice with the *BEIIb* allele (BL06-10), all the rice samples generally had large PV, CPV, SB, CS, and small BD which are characteristic of high amylose rice. Furthermore, these rice samples with the GC allele (BL06 and 07) had larger PV and BD than those with the TT allele. Since SSIIa is generally responsible for the genetic basis of GT, the difference between the RVA profiles might not reflect the allele difference. Among the rice with the *be2b* allele, all the rice materials had small PV, BD, SB, and CS, showing resistance to heat shearing. This is because high amylose may restrict the swelling of starch during heating. Similarly, rice samples with different GC and TT alleles did not show different RVA profiles (Figure 4).

### 3.6. Relationship between Different SSIIa/BEIIb Genotypes

**Figure 5:**
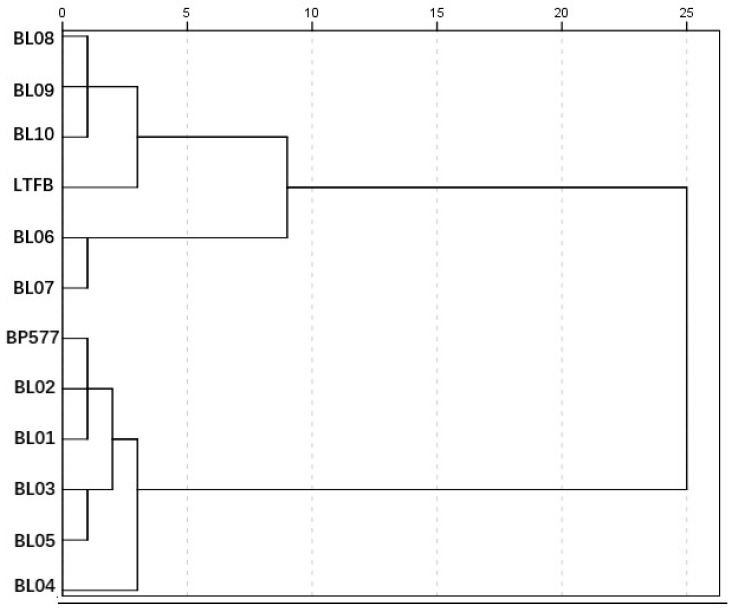
The clustering of genotypes with structural and physicochemical properties.

From the structural and physicochemical properties of the breeding lines derived from the parents BP577 and LTFB with different *SSIIa*-GC/TT and *BEIIb/be2b* alleles, it is clear that rice lines with the *be2b* allele displayed distinct structural and physicochemical properties from those with the *BEIIb* allele. However, in the *be2b* allele background, most parameters between the GC and TT alleles did not show differences, suggesting that the function of SSIIa is not important in the *be2b* allele background. From this aspect, it could be concluded that *BEIIb* is more important in determining the structural and physicochemical properties than *SSIIa*. By analysis of all the BE mutants, Tappiban, et al. [19] also indicated that BEIIb played a more important role in determining the structural and physicochemical properties than BEI and BEIIa. To further reveal the relative important functions of the *SSIIa* and *BEIIb* alleles, clustering analysis was carried out for different genotypes (Figure 5). As expected, two groups were formed as the breeding lines were derived from two parents, BP577 and LTFB. The two groups were formed according to different *BEIIb* alleles but not *SSIIa* alleles, further confirming that BEIIb is more important. For the *BEIIb*-related group (the upper one), rice materials with different GC/TT were revealed in two subgroups, and the parent LTFB was grouped with the BL08-10, indicating that the group represented the low GT with the TT allele. However, for the *be2b*-related group, although two or three subgroups could be revealed, they did not follow the GC/TT groups. Instead, parent BP577, BL01, and BL02 formed a subgroup, BL03 and BL05 formed a second subgroup, and BL04 itself formed a third subgroup (Figure 5).

### 3.7. Relationships between Fine Structure and Physicochemical Properties

To explore the structure-function relationships, the correlation analysis between starch structural parameters and physicochemical properties is shown in Table S2. Most parameters had a significant correlation with each other, except for ΔH, which had no correlation with any other parameters, and breakdown (BD) viscosity, which showed correlations only with some structural parameters. Since there are two types of starch with distinct structural and physicochemical differences, the correlations may also differ from those in previous studies with common starches. The amylose content (AC) was positively correlated with *h_AM_* (r = 0.99, *p* < 0.01) and also had a positive correlation with *X_AP1_, X_AP2_, X_AM_, h_AP2/AP1_*, fb2, and fb3, but a negative correlation with fa and fb1, suggesting the longer B chains and longer amylose chains led to a higher AC. AC is synthesized by the action of GBSS encoded by the *Wx* gene. All the rice materials contain the same *Wx* allele (Figure 1), so the difference in AC was attributed to the deficiency in BEIIb. The deficiency in BEIIb is responsible for the synthesis of larger amylopectin and amylose molecules with longer chains (Table 1) by which the synthesis of short chains of amylopectin was suppressed and the elongation of long chains was promoted [26,37].

BEIIb has an important effect on the crystalline structures of starches by modifying the synthesis of A and B1 chains [43]. The significant changes in the rice amylopectin CLDs in breeding lines with the *be2b* allele modified the RC of the starch granules. RC had a negative correlation with *X_AP1_, X_AP2_, X_AM_, h_AM_, h_AP2/_h_AP1_*, AC, fb3, and $\bar{X}$, but a positive correlation with fa and fb1, which was in agreement with the result of Zhang et al. [26]. The results indicated that lower AC and shorter amylose chain length, or more amylopectin A and B1 chains, would increase the RC.

The 1045/1022 cm^−1^ ratio had a negative correlation with fa (r = 0.92, *p* < 0.01), but had a positive correlation with *X_AP1_, X_AP2_, X_AM_, h_AP2/AP1_*, fb3, and $\bar{X}$ (Table S2), which indicated that longer amylopectin chains can form more double helices and increase the amount of short-range order [26]. RC had a negative correlation with the 1045/1022 cm^−1^ ratio, but a positive correlation with the 1022/995 cm^−1^ ratio (Table S2), which seemed to be contradictory. A possible explanation is that the FTIR data represent the ratio of the proportion of ordered structure to unordered structure, which is irrelevant to long-range order [33].

GT is genetically controlled by SSIIa, whose function is to elongate the short A and B1 chains of amylopectin with DP < 10 to form long B1 chains [15]. T_o_, T_p_, and T_c_ were found to be positively correlated with *X_AP1_, X_AP2_, X_AM_, h_AP2/AP1_*, fb2, fb3, $\bar{X}$, AC, and the 1045/1022 cm^−1^ ratio, but had a negative correlation with RC and the 1022/995 cm^−1^ ratio (Table S2). This result was similar to that of Zhang et al. [26], suggesting these correlations are derived from the *be2b* allele in the rice materials. It should be noted that for the common rice starches, GT generally had a negative correlation with the number of fa chains but a positive correlation with fb1 content [14]. In this study, the correlation between fa and fb1 was positive (r = 0.41, *p* > 0.05), indicating these starches are different from common starches.

The PV, HPV, CPV, SB, and CS had a negative correlation with *X_AP1_, X_AP2_, X_AM_, h_AP2/AP1_*, fb2, fb3, $\bar{X},$ and AC, but a positive correlation with fb1 (Table S2), which is in agreement with previous reports [26,44]. A large amylose chain content, or a large number of long B chains in amylopectin, is expected to extend through crystallites connecting multiple clusters, increasing the integrity of the starch granules, leading to the inhibition of starch swelling [43] and less resistance to shearing.

In conclusion, the structural and physicochemical properties of rice breeding lines changed with different *BEIIb* and *SSIIa* alleles. The *BEIIb*-deficient mutant starches had a higher AC with more amylose long chains, a larger amount of amylopectin long B chains, and a higher degree of molecular order, but a smaller amount of short chains and smaller RC, leading to a higher GT and lower ΔH and pasting viscosities. Therefore, *BEIIb* is more important in determining the structural and physicochemical properties than *SSIIa*. The combination of *be2b*/GC and *be2b*/TT showed no significant difference, suggesting that the function of SSIIa was not important in the *be2b* allele background. The resistant starch content was not measured in this study, but the fact that the *ae* mutant has high resistant-starch content is well known. In the near future, the breeding lines in the background *be2b* will be used for breeding new rice varieties with high resistant-starch content, which will benefit human health.

## Figures and Tables

**Table 1:** Structural parameters of chain length distribution (CLD) of debranched starches obtained from SEC.

Sample	Genotype	*X_AP1_*	*X_AP2_*	*X_AM_*	*h_AM_*	*h_AP2_/h_AP1_*	AC (%)
BP577	GC/*be2b*	17.26 ± 0.10 a	44.04 ± 0.08 ab	729.47 ± 4.76 a	0.42 ± 0.01 cd	0.86 ± 0.00 b	33.16 ± 0.011 bc
LTFB	TT/*BEIIb*	14.09 ± 0.19 c	39.68 ± 0.14 e	580.61 ± 12.17 e	0.25 ± 0.00 ef	0.58 ± 0.00 c	26.85 ± 0.007 e
BL01	GC/*be2b*	17.29 ± 0.07 a	43.89 ± 0.22 bc	710.71 ± 4.62 a	0.40 ± 0.01 d	0.86 ± 0.00 b	32.27 ± 0.002 c
BL02	GC/*be2b*	17.10 ± 0.06 a	43.52 ± 0.15 c	718.95 ± 24.96 a	0.45 ± 0.00 b	0.87 ± 0.00 b	33.84 ± 0.002 ab
BL03	TT/*be2b*	17.19 ± 0.10 a	44.42 ± 0.00 a	704.57 ± 1.52 ab	0.44 ± 0.01 bc	0.89 ± 0.00 a	32.94 ± 0.004 bc
BL04	TT/*be2b*	16.87 ± 0.03 a	43.60 ± 0.22 bc	743.92 ± 3.25 a	0.48 ± 0.02 a	0.88 ± 0.00 ab	34.84 ± 0.009 a
BL05	TT/*be2b*	17.06 ± 0.03 a	43.97 ± 0.15 abc	698.55 ± 7.54 abc	0.42 ± 0.00 bcd	0.86 ± 0.00 b	32.31 ± 0.003 c
BL06	GC/*BEIIb*	15.13 ± 0.06 b	40.50 ± 0.14 d	639.70 ± 2.72 d	0.24 ± 0.01 ef	0.54 ± 0.00 d	28.26 ± 0.005 d
BL07	GC/*BEIIb*	15.16 ± 0.09 b	40.23 ± 0.14 d	642.54 ± 10.95 d	0.22 ± 0.00 f	0.53 ± 0.00 d	26.82 ± 0.000 e
BL08	TT/*BEIIb*	14.65 ± 0.54 b	38.67 ± 0.20 f	651.70 ± 33.37 bcd	0.25 ± 0.00 e	0.53 ± 0.00 d	27.78 ± 0.001 de
BL09	TT/*BEIIb*	14.01 ± 0.00 c	38.60 ± 0.00 f	643.96 ± 41.11 cd	0.25 ± 0.00 e	0.53 ± 0.00 d	27.35 ± 0.003 de
BL10	TT/*BEIIb*	14.09 ± 0.03 c	39.00 ± 0.13 f	617.15 ± 11.76 de	0.24 ± 0.00 ef	0.53 ± 0.01 d	27.18 ± 0.009 de

Values with different letters in the same column are significantly different at *p* < 0.05. *X_AP1_* and *X_AP2_* are the degrees of polymerization (DP) of amylopectin peaks, *X_AM_* represents the DP of amylose peak, *h_AP2/_h_AP1_* is the ratio of the peak heights of amylopectin, *h_AM_* is the peak height of amylose, and AC is amylose content (%).

**Table 2:** Structural parameters of amylopectin CLDs obtained from FACE.

Sample	Genotype	fa	fb1	fb2	fb3	$\bar{X}$
BP577	GC/*be2b*	21.36 ± 0.17 fg	42.53 ± 0.04 f	11.55 ± 0.09 c	24.56 ± 0.05 bc	26.25 ± 0.03 c
LTFB	TT/*BEIIb*	27.27 ± 0.25 c	46.59 ± 0.32 c	11.20 ± 0.07 d	14.95 ± 0.51 ef	21.73 ± 0.22 f
BL01	GC/*be2b*	21.05 ± 0.25 gh	41.74 ± 0.50 g	11.34 ± 0.07 d	25.76 ± 0.82 a	26.94 ± 0.35 a
BL02	GC/*be2b*	21.73 ± 0.21 ef	41.74 ± 0.17 g	11.01 ± 0.04 e	25.52 ± 0.34 ab	26.76 ± 0.15 ab
BL03	TT/*be2b*	20.50 ± 0.08 hi	43.29 ± 0.40 e	12.31 ± 0.11 a	23.90 ± 0.42 c	26.45 ± 0.16 abc
BL04	TT/*be2b*	20.29 ± 0.28 i	43.35 ± 0.07 e	12.01 ± 0.08 b	24.35 ± 0.27 c	26.43 ± 0.11 bc
BL05	TT/*be2b*	22.05 ± 0.61 de	44.06 ± 0.59 d	11.87 ± 0.09 b	21.02 ± 1.11 d	25.34 ± 0.54 d
BL06	GC/*BEIIb*	22.43 ± 0.14 d	52.15 ± 0.34 a	10.69 ± 0.01 fg	14.73 ± 0.47 ef	21.96 ± 0.19 ef
BL07	GC/*BEIIb*	22.09 ± 0.31 de	51.74 ± 0.20 a	10.82 ± 0.13 f	15.35 ± 0.03 e	22.29 ± 0.04 e
BL08	TT/*BEIIb*	29.17 ± 0.24 a	47.68 ± 0.46 b	10.49 ± 0.03 h	12.66 ± 0.73 h	20.57 ± 0.27 h
BL09	TT/*BEIIb*	28.71 ± 0.15 ab	47.46 ± 0.03 b	10.64 ± 0.05 gh	13.19 ± 0.14 gh	20.80 ± 0.08 gh
BL10	TT/*BEIIb*	28.16 ± 0.05 b	47.26 ± 0.08 cb	10.64 ± 0.05 gh	13.93 ± 0.08 gf	21.17 ± 0.03 g

Values with different letters in the same column are significantly different at *p* < 0.05. $\bar{X}$ indicates average chain length.

**Table 3:** Relative crystallinity and IR ratios of rice starches.

Sample	Genotype	RC (%)	1045/1022	1022/995
BP577	GC/*be2b*	21.68 ± 0.33 cd	0.783 ± 0.010 b	0.998 ± 0.011 de
LTFB	TT/*BEIIb*	23.16 ± 0.01 b	0.654 ± 0.005 de	1.060 ± 0.011 a–e
BL01	GC/*be2b*	20.51 ± 0.13 e	0.812 ± 0.005 a	0.937 ± 0.011 e
BL02	GC/*be2b*	20.75 ± 0.27 de	0.782 ± 0.006 ab	1.020 ± 0.011 cde
BL03	TT/*be2b*	21.07 ± 0.16 cde	0.778 ± 0.002 ab	0.925 ± 0.011 e
BL04	TT/*be2b*	20.89 ± 0.34 de	0.753 ± 0.015 bc	0.998 ± 0.011 de
BL05	TT/*be2b*	21.88 ± 0.44 c	0.769 ± 0.001 b	0.967 ± 0.011 de
BL06	GC/*BEIIb*	24.27 ± 0.17 a	0.722 ± 0.026 c	1.102 ± 0.011 a–d
BL07	GC/*BEIIb*	24.22 ± 0.21 a	0.742 ± 0.008 bc	1.042 ± 0.011 b–e
BL08	TT/*BEIIb*	23.38 ± 0.14 ab	0.678 ± 0.007 d	1.164 ± 0.011 ab
BL09	TT/*BEIIb*	23.89 ± 0.25 ab	0.652 ± 0.012 de	1.186 ± 0.011 a
BL10	TT/*BEIIb*	23.09 ± 0.21 b	0.634 ± 0.005 e	1.140 ± 0.011 abc

Values with different letters in the same column are significantly different at *p* < 0.05; RC: relative crystallinity.

**Table 4:** Thermal properties of rice starches.

Sample	Genotype	T_o_	T_p_	T_c_	ΔH
BP577	GC/*be2b*	74.92 ± 0.06 b	83.06 ± 0.58 abc	92.96 ± 0.20 b	8.08 ± 0.24 bcd
LTFB	TT/*BEIIb*	61.24 ± 0.10 e	69.40 ± 0.08 f	77.46 ± 0.06 f	7.14 ± 0.12 cd
BL01	GC/*be2b*	74.73 ± 0.12 b	83.70 ± 0.13 a	93.96 ± 0.00 a	8.96 ± 0.35 ab
BL02	GC/*be2b*	74.01 ± 0.19 c	82.31 ± 0.44 c	92.58 ± 0.68 b	8.37 ± 0.12 abc
BL03	TT/*be2b*	73.74 ± 0.22 c	82.52 ± 0.03 bc	90.43 ± 0.39 c	8.41 ± 0.22 abc
BL04	TT/*be2b*	69.32 ± 0.11 d	80.03 ± 0.07 d	88.99 ± 0.25 d	5.22 ± 0.06 e
BL05	TT/*be2b*	75.52 ± 0.08 a	83.24 ± 0.10 ab	89.82 ± 0.35 cd	6.78 ± 0.52 d
BL06	GC/*BEIIb*	73.66 ± 0.26 c	78.70 ± 0.24 e	83.42 ± 0.28 e	9.38 ± 0.34 a
BL07	GC/*BEIIb*	73.97 ± 0.19 c	78.37 ± 0.16 e	83.67 ± 0.07 e	9.58 ± 1.22 a
BL08	TT/*BEIIb*	58.31 ± 0.06 g	65.04 ± 0.07 g	73.09 ± 0.13 g	6.88 ± 0.07 d
BL09	TT/*BEIIb*	58.24 ± 0.25 g	65.32 ± 0.29 g	73.56 ± 0.46 g	7.27 ± 0.11 cd
BL10	TT/*BEIIb*	58.81 ± 0.00 f	65.58 ± 0.22 g	73.84 ± 0.08 g	6.92 ± 0.03 d

Values with different letters in the same column are significantly different at *p* < 0.05. T_o_: onset temperature; T_p_: peak temperature; T_c_: conclusion temperature; ΔH: enthalpy.

**Table 5:** Pasting properties of rice starches.

Sample	Genotype	PV	HPV	CPV	BD	SB	CS
BP577	GC/*be2b*	115.75 ± 0.00 fg	105.88 ± 0.38 f	142.55 ± 2.13 de	9.88 ± 0.38 cde	26.80 ± 2.13 ef	36.67 ± 1.75 bc
LTFB	TT/*BEIIb*	275.38 ± 6.38 a	246.88 ± 3.63 a	357.29 ± 10.04 a	28.50 ± 2.75 b	81.92 ± 3.67 bc	110.42 ± 6.42 a
BL01	GC/*be2b*	116.71 ± 2.54 fg	110.46 ± 4.79 ef	142.21 ± 13.38 de	6.25 ± 2.25 de	25.50 ± 10.84 ef	31.75 ± 8.59 c
BL02	GC/*be2b*	95.65 ± 4.15 h	89.59 ± 4.67 g	116.75 ± 9.67 e	6.07 ± 0.52 de	21.10 ± 5.52 f	27.17 ± 5.01 c
BL03	TT/*be2b*	126.42 ± 1.09 ef	119.92 ± 0.50 de	158.55 ± 1.38 d	6.50 ± 0.59 de	32.13 ± 2.46 ef	38.63 ± 1.88 bc
BL04	TT/*be2b*	104.46 ± 0.96 gh	100.92 ± 2.25 fg	149.96 ± 3.88 d	3.54 ± 1.29 e	45.50 ± 2.92 de	49.04 ± 1.63 bc
BL05	TT/*be2b*	139.96 ± 0.54 e	131.96 ± 0.13 d	197.09 ± 0.17 c	8.01 ± 0.42 cde	57.13 ± 0.38 d	65.13 ± 0.04 b
BL06	GC/*BEIIb*	231.88 ± 4.63 b	164.71 ± 4.13 c	300.13 ± 8.88 b	67.17 ± 8.75 a	68.25 ± 4.25 cd	135.42 ± 13.00 a
BL07	GC/*BEIIb*	241.50 ± 4.08 b	178.92 ± 4.67 b	306.96 ± 12.04 b	62.59 ± 8.75 a	65.46 ± 7.96 cd	128.05 ± 16.71 a
BL08	TT/*BEIIb*	183.38 ± 7.13 d	166.46 ± 6.96 c	282.71 ± 8.46 b	16.92 ± 0.16 bcd	99.34 ± 1.34 ab	116.25 ± 1.50 a
BL09	TT/*BEIIb*	185.42 ± 3.83 cd	167.79 ± 1.04 bc	294.92 ± 21.75 b	17.63 ± 4.88 bcd	109.51 ± 17.92 a	127.13 ± 22.79 a
BL10	TT/*BEIIb*	199.59 ± 9.58 c	179.67 ± 6.00 b	285.42 ± 0.00 b	19.92 ± 3.59 bc	85.84 ± 9.59 bc	105.75 ± 6.00 a

Values with different letters in the same column are significantly different at *p* < 0.05. PV: peak viscosity; HPV: host paste viscosity; CPV: cold paste viscosity; BD: breakdown; CS: consistency; SB: setback; RVU: Rapid Visco Units.

## Data Availability

Data are contained within the article.

## Supplementary Materials

The following supporting information can be downloaded at: https://www.mdpi.com/article/10.3390/foods12010119/s1, Table S1: The primer sequences; Table S2: Correlation coefficients between starch structural parameters and functional properties.

## Abbreviations

AC	Amylose content
BD	Breakdown
BE	Branching enzyme
CLD	Chain length distribution
CPV	Cold paste viscosity
CS	Consistency viscosity
CEQ	Cooking and eating quality
DP	Degree of polymerization
FACE	Fluorophore-assisted carbohydrate electrophoresis
GT	Gelatinization temperature
HPV	Hot paste viscosity
PV	Peak viscosity
RC	relative crystallinity
RIL	Recombinant inbred line
RVA	Rapid Visco-Analyzer
RVU	Rapid Visco Unit
SB	Setback
SEC	Size-exclusion chromatography
SNP	Single nucleotide polymorphism
SS	Soluble starch synthase
T_c_	conclusion temperature
T_o_	Onset temperature
T_p_	peak temperature
*Wx*	Waxy
ΔH	Enthalpy of gelatinization
