# Supplementary material for: Combined Effects of BEIIb and SSIIa Alleles on Amylose Contents, Starch Fine Structures and Physicochemical Properties of Indica Rice
Source: Foods. 2022 Dec 26;12(1):119. doi: 10.3390/foods12010119 (PMC9818509; doi:10.3390/foods12010119)
Supplement: Supplementary file 1 [file foods-12-00119-s001.zip › foods-2045256-supplementary.pdf]

Combined effects of *BEIIb* and *SSIIa* alleles on amylose contents, starch fine structures and physicochemical properties of indica rice

Yaqi Hu [1,2], Yanni Zhang[1], Shouwu Yu[3], Guofu Deng [4], Gaoxing Dai [4*], Jinsong Bao [1, 2*]

[1] Institute of Nuclear Agricultural Sciences, College of Agriculture and Biotechnology, Zhejiang University, Zijingang Campus, Hangzhou, 310058, China

[2]Hainan Institute of Zhejiang University, Yazhou Bay Science and Technology City, Yazhou District, Sanya 572025, China

[3]Zhejiang Academy of Agricultural Sciences, No.198,Shiqiao Road Shangcheng District, Hangzhou, China.

[4]Rice Research Institute, Guangxi Academy of Agricultural Sciences, Nanning, China

*Corresponding authors: jsbao@zju.edu.cn (J.B.); 25266220@qq.com (G.D.);Tel: +86-571-86971932 (J.B.)

Table S1. The primer sequences.

Gene	Forward primer (5' → 3')	Reverse primer (5' → 3')
BEIIb	TGCACCAAGTAGTCGTTTCGGG	TGAGCAGAGGGAGTAATTAATGA
SSIIa	CGAGGCGCAGCACAACAG	GGCCGTGCAGATCTTAACCAT
	CAAGGAGAGCTGGAGGGGGC	ACATGCCGCGCACCTGGAAA
Wx	CTTTGTCTATCTCAAGACAC	TTGCAGATGTTCTTCCTGATG

Table S2. Correlation coefficients between starch structural parameters and functional properties.

	X_{AP1}	X_{AP2}	X_{AM}	h_{AM}	*hap2/hap1*	AC	fa	fb1	fb2	fb3	$\bar{X}$	RC	1045/1022	1022/995	To	Tp	Tc	ΔH	PV	HPV	BD	CPV	SB
X_{AP2}	**0.98****																						
X_{AM}	**0.92****	0.86**																					
h_{AM}	**0.92****	0.93**	0.91**																				
hap2/hap1	0.95**	0.97**	0.88**	0.98**																			
AC	0.94**	0.93**	0.94**	0.99**	0.97**																		
fa	-0.88**	-0.89**	-0.75**	-0.73**	-0.77**	-0.77**																	
fb1	-0.74**	-0.76**	-0.72**	-0.87**	-0.88**	-0.83**	0.41																
fb2	0.75**	0.84**	0.64*	0.81**	0.83**	0.77**	-0.70*	-0.64*															
fb3	0.96**	0.97**	0.88**	0.95**	0.98**	0.95**	-0.83**	-0.85**	0.76**														
$\bar{X}$	0.97**	0.98**	0.88**	0.95**	0.98**	0.95**	-0.86**	-0.82**	0.78**	1.00**													
RC	-0.85**	-0.86**	-0.81**	-0.94**	-0.94**	-0.91**	0.62*	0.94**	-0.72**	-0.94**	-0.92**												
1045/1022	0.95**	0.91**	0.83**	0.78**	0.84**	0.81**	-0.92**	-0.57	0.62*	0.89**	0.90**	-0.72**											
1022/995	-0.88**	-0.92**	-0.66*	-0.78**	-0.87**	-0.76**	0.87**	0.63*	-0.85**	-0.87**	-0.90**	0.77**	-0.87**										
To	0.83**	0.82**	0.64*	0.60*	0.68*	0.64*	-0.94**	-0.30	0.55	0.73**	0.77**	-0.48	0.92**	-0.81**									
Tp	0.92**	0.92**	0.75**	0.75**	0.81**	0.78**	-0.97**	-0.48	0.68*	0.86**	0.88**	-0.65*	0.96**	-0.89**	0.98**								
Tc	0.96**	0.96**	0.82**	0.84**	0.89**	0.86**	-0.94**	-0.64*	0.69*	0.94**	0.95**	-0.78**	0.97**	-0.90**	0.92**	0.98**							
ΔH	0.11	0.07	-0.09	-0.22	-0.10	-0.17	-0.31	0.29	-0.23	0.03	0.05	0.18	0.36	-0.18	0.51	0.36	0.28						
PV	-0.84**	-0.79**	-0.94**	-0.91**	-0.87**	-0.92**	0.56	0.84**	-0.57	-0.85**	-0.83**	0.87**	-0.70*	0.56	-0.45	-0.59*	-0.71*	0.19					
HPV	-0.87**	-0.80**	-0.97**	-0.86**	-0.82**	-0.90**	0.70*	0.68*	-0.50	-0.84**	-0.83**	0.77**	-0.80**	0.57	-0.62*	-0.71*	-0.78**	-0.01	0.95**				

BD	-0.50	-0.51	-0.59*	-0.73**	-0.69*	-0.66*	0.10	0.91**	-0.52	-0.61*	-0.57	0.80**	-0.28	0.36	0.04	-0.15	-0.32	0.56	0.78**	0.55			
CPV	-0.93**	-0.90**	-0.95**	-0.95**	-0.93**	-0.96**	0.72**	0.85**	-0.64*	-0.95**	-0.93**	0.92**	-0.83**	0.71**	-0.63*	-0.75**	-0.85**	0.03	0.97**	0.95**	0.68*		
SB	-0.92**	-0.93**	-0.78**	-0.82**	-0.87**	-0.84**	0.89**	0.68*	-0.65*	-0.94**	-0.95**	0.82**	-0.91**	0.88**	-0.84**	-0.91**	-0.96**	-0.30	0.70*	0.76**	0.36	0.86**	
CS	-0.90**	-0.91**	-0.84**	-0.95**	-0.96**	-0.93**	0.67*	0.94**	-0.72**	-1.00**	-0.95**	1.00**	-0.78**	0.80**	-0.57	-0.72**	-0.84**	0.07	0.89**	0.81**	0.76**	0.95**	0.88**

* and ** indicate significant at $p < 0.05$ and 0.01 levels, respectively.
